# Early recurrence of atrial arrhythmia following catheter ablation of atrial tachycardia consecutive to ablation of atrial fibrillation using the updated blanking period

**DOI:** 10.1016/j.hroo.2025.12.003

**Published:** 2025-12-13

**Authors:** Raphael Spittler, Antonia Dalmer, Karoline Linke, Alexandra Marx, Hanke Mollnau, Blanca Quesada-Ocete, Peter Seidel, Bernard Prengel, Emin S. Gezinir, German Fernandez Ferro, Alexander P. Benz, Thomas Rostock

**Affiliations:** 1 Department of Cardiology II – Electrophysiology, University Hospital Mainz, Mainz, Germany; 2 Population Health Research Institute, McMaster University, Hamilton, Canada

**Keywords:** Atrial tachycardia, Arrhythmia recurrence, Blanking period, Early recurrence, Ablation, Atrial fibrillation

## Abstract

**Background:**

In patients undergoing catheter ablation of atrial fibrillation (AF), arrhythmia recurrence during the blanking period is usually not considered a failure of treatment. The prognostic significance of early recurrence (ER) of atrial arrhythmia after ablation of atrial tachycardia (AT) subsequent to AF ablation is less certain.

**Objective:**

This study aimed to explore the association between early and late recurrence of atrial arrhythmia after ablation of AT subsequent to AF ablation.

**Methods:**

Patients undergoing a first ablation of AT subsequent to AF ablation between 2015 and 2021 were included in this retrospective, single-center, observational cohort study. Recurrent atrial arrhythmia was defined as AT or AF lasting more than 30 seconds. Regression models were built to assess the association between early (within 56 days) and late (beyond 56 days) recurrence of AT.

**Results:**

A total of 194 patients were included (mean age 67 ± 10 years, 63% male), of whom 71 (37%) had pulmonary vein isolation only, and 123 (63%) had received complex AF ablation during their index procedure. Fifty-two patients (27%) had ER of atrial arrhythmia. During a mean follow-up of 23.6 ± 15.5 months, 118 patients (61%) had late recurrence of atrial arrhythmia (111 with AT, 7 with AF). ER was associated with late recurrence (hazard ratio 1.53; 95% confidence interval 1.05–2.34; *P* = .02).

**Conclusion:**

One in 4 patients undergoing catheter ablation of AT subsequent to AF ablation had recurrent atrial arrhythmia within 56 days after ablation. ER was associated with late recurrence of atrial arrhythmia.

## Graphical abstract

**Figure:**
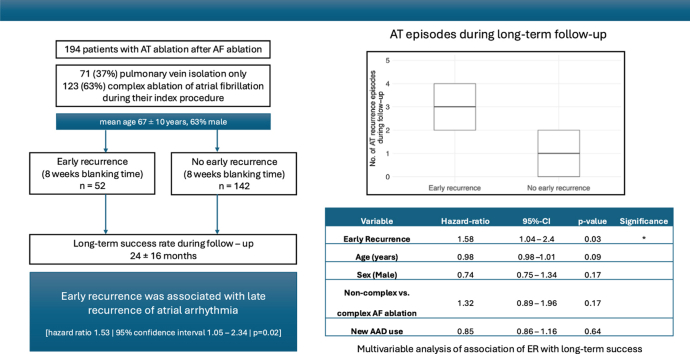

**Key Findings**

▪ Early recurrence within the first 56 days after atrial tachycardia ablation performed for post–atrial fibrillation ablation was independently associated with late recurrence of atrial arrhythmia in both univariable and multivariable analyses.
▪ Patients with early recurrence experienced a higher number of arrhythmia episodes beyond 56 days.
▪ Early recurrence demonstrated moderate predictive value for late arrhythmia recurrence (positive predictive value 69%), whereas the absence of early recurrence did not reliably exclude future arrhythmia recurrence (negative predictive value 47%).

## Introduction

Early recurrence (ER) of arrhythmia after atrial fibrillation (AF) ablation has traditionally not been considered a failure of treatment. Potential reasons for ER include a transient inflammatory response accompanying the destruction of myocardial tissue. This understanding has led to the introduction of a “blanking” period subsequent to AF ablation in numerous studies.^1^ Current guidelines recommend a 56-day blanking period after AF ablation.^2^

However, emerging evidence suggests that ER after AF ablation may carry prognostic significance, prompting a re-evaluation of the traditional concept of the blanking period and its clinical implications.^3^, ^4^, ^5^

The prognostic significance of ER of atrial arrhythmia after ablation of atrial tachycardia (AT) subsequent to AF ablation is less certain. We aimed to explore the association between early and late recurrence of AT/AF after ablation of AT subsequent to AF ablation.

## Methods

### Study population

We performed a retrospective chart review of patients undergoing their first AT ablation subsequent to AF ablation at the University Medical Center Mainz between March 2015 and June 2021. We only included patients with 1 subsequent AT ablation after mainly 1 with a maximum of 2 previous AF ablation (index procedure). Patients with more previous AF ablation procedures were excluded. We excluded patients with an index procedure done primarily for an arrhythmia other than AF and patients with a follow-up time of less than 90 days after the study-qualifying AT ablation. The study was approved by the local ethics committee and adhered to the Declaration of Helsinki. An informed consent was obtained from all participants.

### Outcome definition

ER of arrhythmia was defined as AT/AF within 56 days after ablation of AT. Late recurrence was defined as any arrhythmia beyond the first 56 days after ablation of AT.

### Electrophysiological procedure and catheter ablation

The index procedure and the AT ablation procedure were all performed using radiofrequency (RF) ablation using conventional energy settings (25–35 W); thus, no single-shot device or high-power short-duration approach was used.

Details of the procedural approach have been described previously.^6^ In brief, a steerable decapolar catheter (Inquiry, IBI, Irvine, CA) was placed in the coronary sinus via femoral vein access. Until 2019, a circumferential decapolar catheter (Lasso; Biosense Webster, Irvine, CA) with the Ensite NavX system (Abbott, Abbott Park, IL) was used. From 2019 onward, mapping was performed using the PENTARAY NAV ECO high-density catheter (Biosense Webster) with the CARTO 3 system (Biosense Webster). In all patients, ablation was conducted using a 3.5 mm irrigated-tip catheter with contact force (THERMOCOOL SMARTTOUCH SF, Biosense Webster). We used 30–35 W at the anterior wall or at the roof and 25 W at the posterior wall and the dragging approach using the NavX system and the ablation index (posterior 400 and anterior 550) when using the CARTO system. Mapping of ATs was performed with conventional methods, such as entrainment and local activation mapping (relative to fixed intracardiac electrogram and/or to P-wave onset) and using 3-dimensional electroanatomic mapping systems (Ensite NavX and CARTO 3 mapping systems). First, we evaluated the cycle length regularity. In the case of a regular cycle length, a macro-reentrant mechanism was evaluated using local activation sequences at different left atrial (LA) areas (coronary sinus, LA appendage, anterior, posterior, and lateral LA). In addition, entrainment mapping was performed at the same LA sites. Assuming a focal AT mechanism, a detailed electroanatomic activation mapping targeting the earliest endocardial activation was performed (Supplemental Figure 1).

In all cases, the initial step during the study-qualifying procedure was the reisolation of the pulmonary veins if required, after AT mechanism analysis. If previous procedures involved left linear or right atrial isthmus ablations, bidirectional block was assessed, and if incomplete, further ablation was performed to restore a full block. Bidirectional block was verified by differential pacing; thus, pacing was performed with the ablation catheter positioned near and distant from the line, while measuring conduction delay to a second diagnostic catheter on the opposite side, and vice versa. In later cases, we additionally used high-resolution activation mapping with pacing in the LA appendage using a high-density mapping catheter.

The procedural endpoint included termination of the clinical AT and noninducibility of any other AT.

### Follow-up

All patients underwent systematic follow-up at our outpatient clinic after 6 and 12 months, including 12-lead electrocardiogram (ECG) and detailed history, supplemented by 24-hour Holter ECGs and 12-lead ECGs from referring physicians, as well as structured telephone interviews after the 12-month systematic follow-up. Any documented atrial arrhythmia lasting over 30 seconds on 24-hour Holter or 12-lead ECG was considered a recurrence, with additional examinations performed in the case of symptoms suggestive of arrhythmia. Any arrhythmia occurring during the follow-up period was documented, including the date, frequency, underlying mechanism (categorized as AT or AF), and corresponding therapies. The use of antiarrhythmic drugs (AADs) was documented and compared at the time of the procedure and the last follow-up.

### Statistical analysis

We descriptively analyzed baseline characteristics of all patients included and separately of those with and without early arrhythmia recurrence, respectively. Continuous variables were summarized as mean with standard deviation or median (interquartile range), as appropriate. Categorical variables were summarized as counts and percentages. The proportion of patients with early and late recurrence of atrial arrhythmia was descriptively analyzed. The association between early and late recurrence was studied using Cox proportion hazard models, and results reported using a hazard ratio with 95% confidence interval. Kaplan-Meier analysis was used for visualization. All significance tests were 2 tailed with rejection of the null hypothesis at *P* < .05. Statistical analysis was performed with R (R Foundation for Statistical Computing, R Development Core Team, Vienna, Austria).

## Results

### Study population

**Figure 1:**
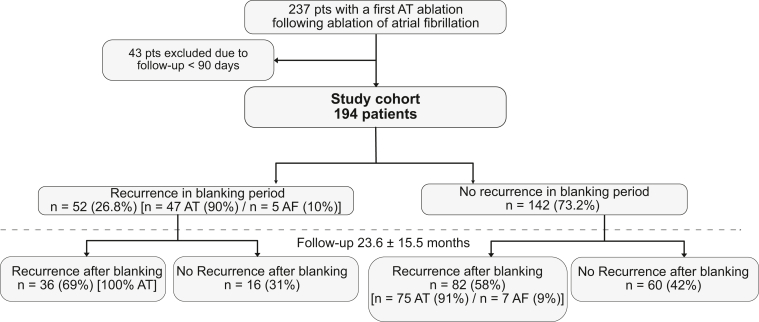
Derivation of study population. Blanking period was defined as the time interval 56 days after the procedure. AT = atrial tachycardia.

We included 194 patients who underwent 1 AT ablation of AT subsequent to almost 1 AF ablation (1 pre-ablation n = 173 (89%); 2 pre-ablations n = 21 [11%]) (Figure 1). Overall, the mean age was 67 ± 10 years, and 63% were male (Table 1) with a mean body mass index of 27.2 ± 3.7 kg/m^2^. Mean LA volume was 69.8 ± 26.9 mL, and moderate or severe mitral regurgitation was diagnosed in 26 patients (13%). Furthermore, 42 patients (22%) had coronary heart disease, 143 had arterial hypertension (74%), and 20 had diabetes (10%). Overall, 50 patients (26%) were on an AAD at baseline.

**Table 1:** Baseline characteristics

Parameter	Study cohort (N = 194)
Age in y, mean ± SD	67 ± 10
Male sex, n (%)	122 (63)
BMI in kg/m^2^, mean ± SD	27.2 ± 3.7
LA volume in mL, mean ± SD	69.8 ± 26.9
Details of index ablation for AF	
Previous PVI, n (%)	18 (9)
Previous PVI + CTI, n (%)	9 (5)
Previous complex ablation procedure, n (%)	123 (63)
PVI with PV trigger n (%)	44 (23)
Chronic heart failure, n (%)	12 (6)
Hypertension, n (%)	143 (74)
Diabetes mellitus, n (%)	20 (10)
Previous stroke, n (%)	16 (8)
CHA_2_DS_2_-VASc score, mean ± SD	2.8 ± 1.6
Coronary artery disease, n (%)	42 (22)
Antiarrhythmic drug	
Flecainide, n (%)	10 (5)
Amiodarone, n (%)	35 (18)
Dronedarone, n (%)	4 (2)
Propafenone, n (%)	1 (1)
Beta-blocker, n (%)	151 (78)

AF = atrial fibrillation; BMI = body mass index; CTI = cavotricuspid isthmus; LA = left atrial; PV = pulmonary vein; PVI = pulmonary vein isolation; SD = standard deviation.

Of all patients included, 62 patients (32%) had undergone pulmonary vein isolation (PVI) only, 9 patients (5%) PVI and ablation of the cavotricuspid isthmus, and 123 patients (63%) complex LA ablation during their index procedure for AF ablation. Complex ablation procedures consisted in all cases of PVI, mitral isthmus line ablation in 50 of the 123 cases (41%), anterior line ablation in 15 of the cases (12%), roof line in 60 of the cases (49%), and additional substrate modification targeting complex fractionated electrograms and low-voltage areas in 108 patients (88%).

### Catheter ablation of AT

At the beginning of the study-qualifying procedure, 142 patients (73%) were in AT (Table 2). The primary mechanism of clinical AT was diagnosed as macro-reentry in 116 patients (60%), as focal in 59 (30%), and as localized reentry in 19 (10%). Both focal and macro-reentry AT were found in 15 patients (7%), whereas 4 patients (2%) had macro-reentry and localized reentry ATs, and 2 patients (1%) exhibited all 3 types. In addition, 19 patients (10%) presented with pulmonary vein-dependent ATs. The clinical AT was limited to the LA in 158 cases (81%) and to the right atrium in 21 cases (11%). A biatrial mechanism was found in the remaining 15 patients (8%). Left macro-reentry tachycardias were found in 74 patients (38%).

**Table 2:** Catheter ablation of AT

Parameter	Study cohort (N = 194)
Total procedure time in min, mean ± SD	109 ± 38
Fluoroscopy time in min, mean ± SD	14.5 ± 9.6
Rhythm at baseline	
Clinical AT, n (%)	142 (73)
AF, n (%)	4 (2)
Sinus rhythm, n (%)	48 (25)
Mechanism of clinical AT	
Macro-reentrant, n (%)	116 (60)
Focal, n (%)	59 (30)
Localized reentry, n (%)	19 (10)
Localization	
Left, n (%)	158 (81)
Right, n (%)	21 (11)
Biatrial, n (%)	15 (8)
Ablation	
Pulmonary vein reisolation, n (%)	68 (35)
Mitral isthmus line,∗ n (%)	63 (32)
Anterior line,† n (%)	49 (25)
Roof line,‡ n (%)	70 (48)
Cavotricuspid isthmus,§ n (%)	50 (25)
Substrate modification in the previous procedure, n (%)	108 (56)

AF = atrial fibrillation; AT = atrial tachycardia; CTI = cavotricuspid isthmus; SD = standard deviation.

∗ Of 49 anterior line ablations, 41 were de novo and 8 had reablation of previously applied linear ablation, and 7 had persistent anterior line block from a previous AF procedure.

† Of 63 mitral isthmus line ablations, 41 were de novo and 22 had reablation of previously applied mitral isthmus ablation, and 28 had persistent mitral isthmus block from a previous AF procedure.

‡ Of 70 roof line ablations, 52 were de novo and 18 had reablation of previously applied roof line ablation, and 42 had persistent roof line block from a previous AF procedure.

§ Of 50 CTI ablations, 33 were de novo and 17 had reablation of previously applied roof line ablation, and 21 had persistent CTI block from a previous AF procedure.

A total of 68 patients (35%) had reconnection of at least 1 pulmonary vein and underwent reisolation. De-novo mitral isthmus line ablation was performed in 41 patients (21%), roof line ablation in 52 (27%), and anterior line in 41 patients (21%). Reablation of a mitral isthmus line applied during the index procedure for AF was performed in 22 patients (11%), of the roof line in 18 patients (9%), and of the anterior line in 8 patients (4%).

In 32 patients (16%), the clinical AT was diagnosed as common-type right atrial flutter and subsequently underwent cavotricuspid isthmus ablation. Electrical recovery of the cavotricuspid isthmus was observed in 17 patients (9%).

Ultimately, intraprocedural termination of the AT was achieved in 191 patients (98%). The mean total procedure time was 109 ± 38 minutes.

### Recurrent atrial arrhythmia

**Figure 2:**
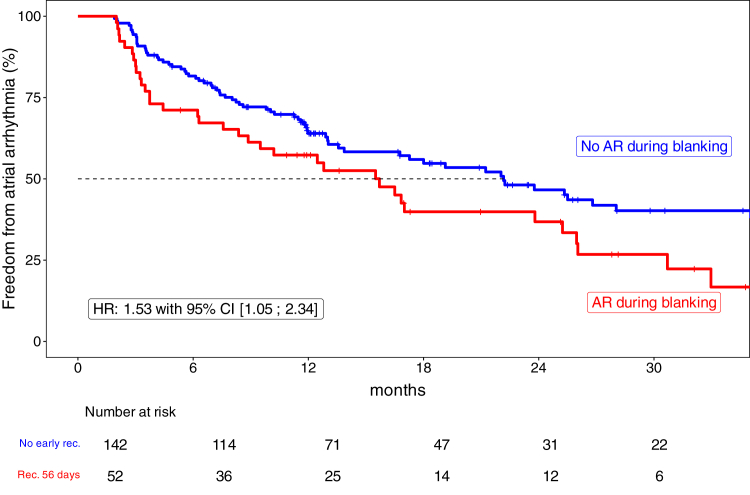
Recurrent atrial arrhythmia beyond 56 days after ablation. The Kaplan-Meier curve shows freedom from recurrent atrial arrhythmia beyond 56 days in patients with and without early recurrence. Clinical outcome after atrial fibrillation ablation (89% 1 and 11% 2 preprocedures) and subsequent atrial tachycardia ablation. The figure is truncated at 36 months. AR = arrhythmia recurrence; CI = confidence interval; HR = hazard ratio.

A total of 52 patients (26.8%) had ER of atrial arrhythmia during the first 56 days after ablation (47 patients [90%] AT and 5 patients [10%] AF recurrence) (Figure 2). Baseline characteristics of patients with and without ER of atrial arrhythmia are presented in Supplemental Table 1. Procedural details of patients with and without ER are presented in Supplemental Table 2.

During a median follow-up time of 18.5 months [interquartile range 6.3–63.2] beyond 56 days after ablation, 118 patients (61%) had late recurrence of atrial arrhythmia. Of them, 111 patients (94%) had AT, and 7 patients (6%) had AF recurrence.

ER within 56 days after ablation was associated with late recurrence (hazard ratio 1.53; 95% confidence interval 1.05–2.34; *P* = .02) (Figure 2). Analyses adjusted for age, sex, new AAD use, and type of index procedure (noncomplex only vs complex) yielded consistent results (Supplemental Table 3).

**Figure 3:**
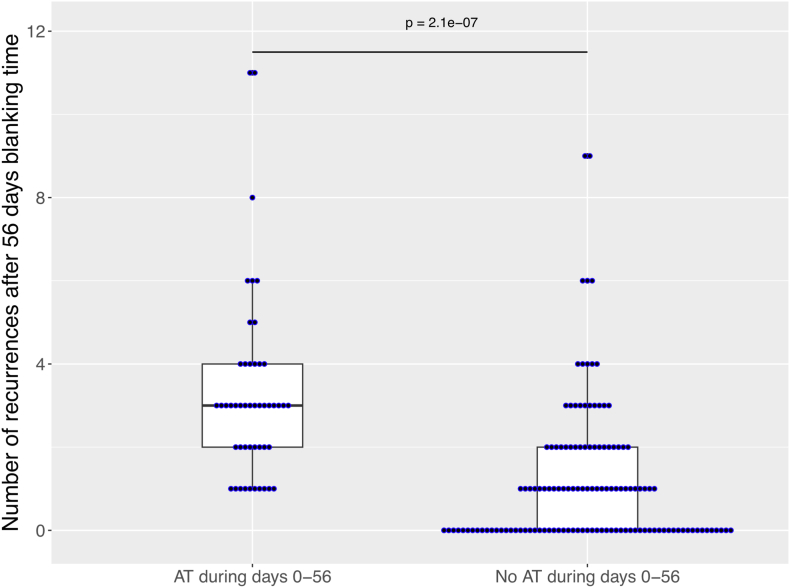
Number of episodes of recurrent atrial arrhythmia beyond 56 days after ablation. The figure shows box plots summarizing the distribution of the number of episodes of recurrent atrial arrhythmia beyond 56 days in patients with and without early recurrence. Patients with early recurrence had a median (interquartile range [IQR]) number of 3 episodes (2–4), and patients without early recurrence had a median (IQR) number of 1 episode (0–2) beyond 56 days after ablation. The box plot displays the distribution of data by showing the median (*central line*), IQR (*box*), and potential outliers (*points outside the whiskers*). The *box* spans from the 25th to the 75th percentile, whereas the *whiskers* extend to the minimum and maximum values within 1.5 times the IQR. Every *blue dot* represents the arrhythmia recurrence number of 1 patient. AT = atrial tachycardia.

In more detail, 52 patients with subsequent AT ablation after AF ablation had an ER during the blanking period. Of them, 36 (69%) had an additional arrhythmia recurrence (only AT) during the follow-up after 56 days. In contrast, only 82 patients (75 AT [91%] and 7 [9%] AF) of the remaining 142 patients without an arrhythmia recurrence during the blanking period had arrhythmia recurrence during follow-up. Moreover, patients with an arrhythmia recurrence during the blanking period had a higher number of arrhythmia recurrences (2.3 ± 2.2 vs 1.2 ± 1.6; *P* < .001), resulting in more electrical cardioversion needed during follow-up (1.2 ± 1.6 vs 0.6 ± 1.3; *P* < .001) (Figure 3).

To evaluate the association between ER and arrhythmia recurrence during follow-up, we calculated key diagnostic performance metrics. The sensitivity was 32.4%, and the specificity was 80.7%, resulting in a positive predictive value of 69% and a negative predictive value of 47%.

### Antiarrhythmic drug therapy

At the time of the AT ablation procedure, 50 patients (26%) were under AAD therapy. Of them, 35 (18%) were treated with amiodarone, 4 (2%) with dronedarone, 10 (5%) with flecainide, and 1 (1%) with propafenone. After the last follow-up, 28 patients (14%) were under AAD therapy, and 17 of them (9%) were treated with amiodarone, 3 (2%) with dronedarone, 6 (3%) with flecainide, 1 (1%) with propafenone, and 1 (1%) with verapamil.

## Discussion

We found that approximately 1 in 4 patients experienced ER of atrial arrhythmia after catheter ablation of AT subsequent to AF ablation. ER within 56 days was associated with a higher risk of late recurrence of atrial arrhythmia, and these patients experienced more arrhythmic episodes beyond the 56-day blanking period. In contrast, the absence of ER was a poor predictor of freedom from late arrhythmia. These findings suggest that although the presence of ER is a moderate predictor of subsequent recurrence, its absence does not reliably exclude the possibility of late atrial arrhythmia. Complex AF ablation procedures could facilitate complex and difficult-to-map AT circuits. Although two-thirds of our cohort underwent such a complex ablation procedure, ER remained the only variable associated with late recurrence in a multivariable Cox regression model with noncomplex vs complex AF ablation.

A “blanking period” after AF ablation was introduced to account for transient inflammation and edema, both of which may contribute to AF recurrence. This “blanking period” was originally defined as the initial 3 months after ablation in which arrhythmia recurrences were not considered prognostically relevant for the long-term outcome. However, recent studies have questioned both the duration and the prognostic relevance of the “blanking period.” Darden et al^7^ described an association between early and late AF recurrence in a cohort of 259 patients, particularly within the first 4 weeks. Similarly, Kahle et al^8^ observed higher rates of repeat ablations and late recurrences in patients with ERs.

Their study analyzed ER within the first 3 months, whereas our analysis focused on ER occurring within the first 56 days. Recurrence rates were comparable in their study, but only two-thirds of their patients experienced AT compared with a higher proportion in our cohort. After adjustment for age, sex, and PVI, only the hazard ratio in our study remained lower than in the study by Kahle et al.^8^ Overall, our findings confirm that the association between early and late recurrence remains evident even when applying the shorter blanking period recommended by recent guidelines.

The appropriate treatment of ERs after AT ablation can be challenging owing to early relapses after cardioversion and the existence of persistent edema and inflammation of the arrhythmia substrate.

Given that arrhythmia recurrences in our cohort were predominantly AT (>90%) and more than half of patients with recurrence beyond 56 days experienced only 1 recurrence during long-term follow-up, our findings support cardioversion as the preferred initial therapeutic approach.

Patients with ER within 30 days after AT ablation had only 1 recurrence in 20% of cases, again favoring electrical cardioversion as the early treatment of AT recurrences.

Supporting these findings, Derval et al^9^ found AT recurrence in 46% of patients after complex AF procedures, similar to our cohort’s 51% recurrence rate after AT ablation. Derval et al^9^ found in their cohort with high-density mapping that only 8% had true focal AT. The higher prevalence of “focal” AT in our cohort could be a mismatch with epicardial circuits or localized reentries owing to the use of conventional mapping techniques.

Patients with early AT recurrences had a higher probability of subsequent AT and a higher incidence of any arrhythmia. This observation has also been reported by Choi et al.^10^ Furthermore, Andrade et al^11^ reported that 50% of patients with early AT recurrence experienced no further arrhythmias after the first 3 months after ablation.

The mechanisms underlying recurrence of AT and AF after ablation differ significantly, both in terms of their origin and the implications for treatment. AT recurrences after complex ablation with concomitant linear formation and substrate modification often indicate lesion-related conduction gaps or reentrant channels formed during or after the initial AF ablation procedure.^9^ This may warrant more precise mapping or additional ablation of arrhythmogenic isthmuses or foci.^12^

In contrast, AF recurrences, particularly in the presence of persistent PVI, are more likely to result from more diffuse changes in the atrial substrate, including fibrosis or electrical remodeling. Thus, they are less likely to be resolved by targeting specific regions.^13^

The recurrence rate in our cohort is comparable with studies by Luik et al^14^ with a multiprocedural success rate of 55% for any arrhythmia after 30-month follow-up or with the study by Takigawa et al^15^ with a recurrence rate of 46.3% after a mean time of 4 months after a single AT ablation in a cohort of AF ablation–related ATs. Nevertheless, the success rate is far beyond the desired outcome, and future ablation approaches should address this issue with new emerging technologies such as pulsed field ablation, which is a promising approach for linear lesion and selected focal ATs. However, head-to-head studies have not shown superior arrhythmia-free survival vs RF for linear lesions and long-term data specific to post-AF ablation of ATs remain limited. Vein of Marshal ablation as an adjunctive therapy can facilitate achieving durable mitral isthmus block, which has been shown in randomized and observational data to increase freedom from arrhythmia recurrence.^16^

### Limitations

Our study has several limitations. First, this was a single-center retrospective analysis with a modest sample size. Second, recurrent atrial arrhythmia after ablation of AT was detected using intermittent Holter monitoring or when diagnosed clinically. Thus, reported rates of atrial arrhythmia recurrences are likely underestimated, and we are unable to report on AT/AF burden. Third, these findings primarily apply to thermal (ie, RF) ablation. Fourth, owing to the use of conventional AT mapping techniques, we cannot exclude a mismatch of ATs with centrifugal activation, given that they may represent localized reentries (including epicardial circuits). Finally, we are unable to assess the relationship between early and late recurrence on quality of life and health care utilization, and owing to the limited number of recurrence events during the first 90 days after the AT ablation, it was not possible to exactly calculate a cutoff to define an optimal duration for the blanking period.

## Conclusion

In this study, 1 in 4 patients undergoing catheter ablation of AT subsequent to AF ablation had recurrent atrial arrhythmia within 56 days after ablation. ER was associated with late recurrence of atrial arrhythmia, and patients with ER experienced more episodes of recurrent arrhythmia beyond 56 days.

## Disclosures

T.R. received a speaking fee from Biosense Webster. R.S. was supported with a travel grant by Biosense Webster and received a speaking fee from Bristol Myers Squibb. All other authors have nothing to disclose.

## Footnotes

Appendix Supplementary data associated with this article can be found in the online version at https://doi.org/10.1016/j.hroo.2025.12.003.
