# Supplementary figures and images for: Early recurrence of atrial arrhythmia following catheter ablation of atrial tachycardia consecutive to ablation of atrial fibrillation using the updated blanking period
Source: Heart Rhythm O2. 2025 Dec 13;7(2):248–54. doi: 10.1016/j.hroo.2025.12.003 (PMC12925935; doi:10.1016/j.hroo.2025.12.003)

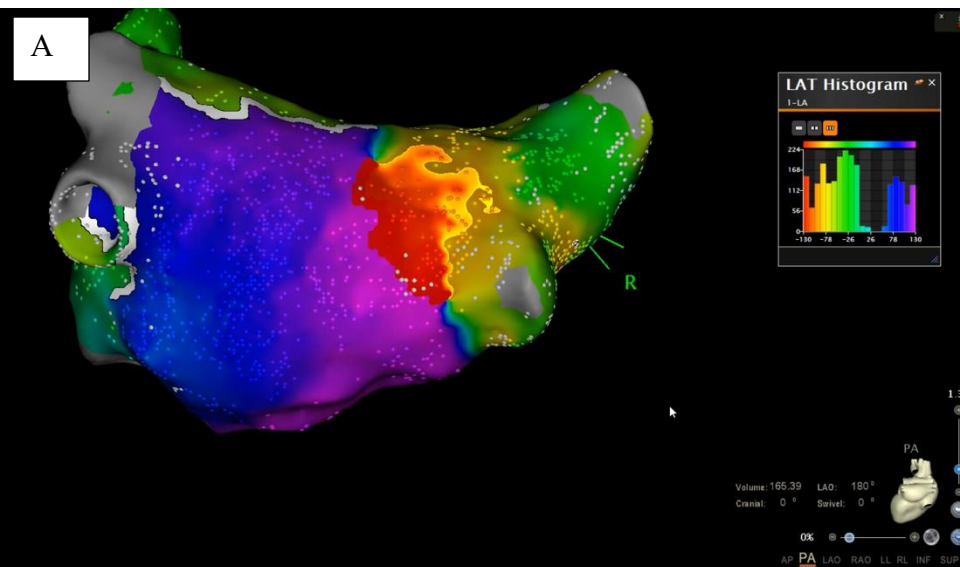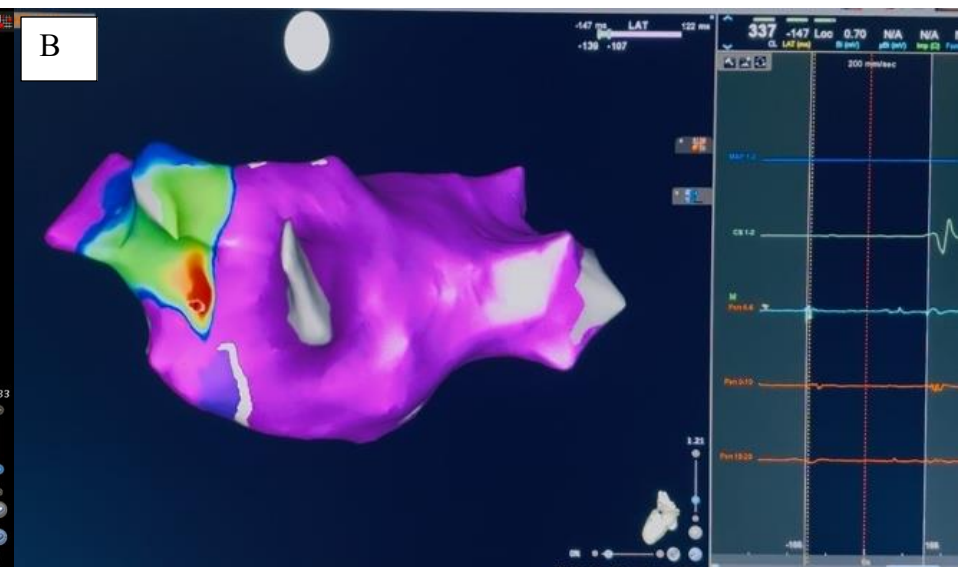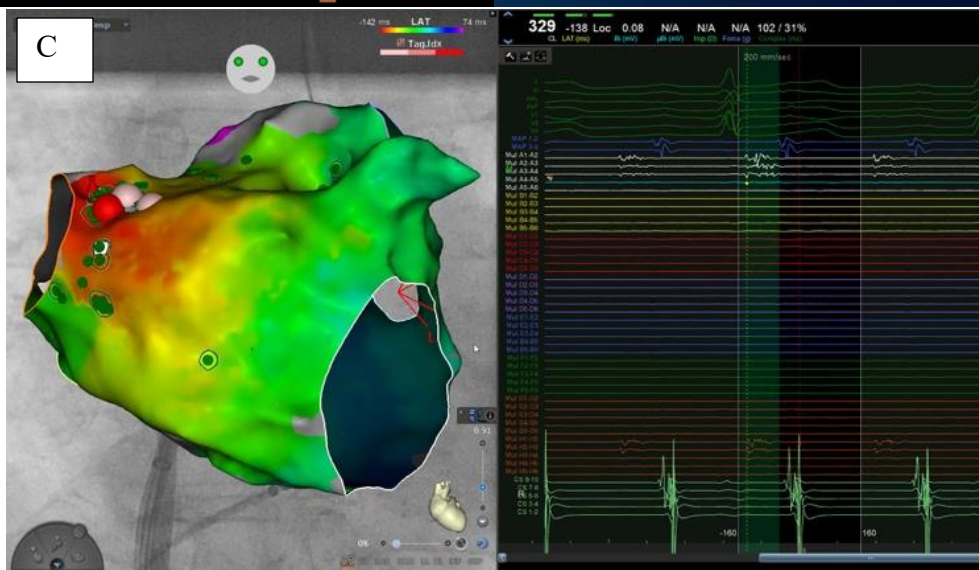

Supplement: Supplementary Figure [file mmc1.pdf]
