# Supplementary material for: Early recurrence of atrial arrhythmia following catheter ablation of atrial tachycardia consecutive to ablation of atrial fibrillation using the updated blanking period
Source: Heart Rhythm O2. 2025 Dec 13;7(2):248–54. doi: 10.1016/j.hroo.2025.12.003 (PMC12925935; doi:10.1016/j.hroo.2025.12.003)
Supplement: Supplementary Material [file mmc2.docx]

**Supplement Table 1: Baseline characteristics of patients with an early recurrence vs. patients without an early recurrence**

| Baseline characteristics (N= 194) | Pts. with ER (N=52) | Pts. without ER (N=142) | p |
| --- | --- | --- | --- |
| Age in years, mean ± SD | 67.71 ± 9.58 | 67.38 ± 10.28 | 0.9 |
| Male sex, n (%) | 33 (63) | 89 (63) | 1 |
| BMI in kg/m2, mean ± SD (n) | 27.36 ± 4.37 | 27.18 ± 3.43 | 0.6 |
| LA volume in mL, mean ± SD (n) | 70.54 ± 25.56 | 69.5 ± 27.47 | 0.6 |
| Details of index ablation for AF |  |  |  |
| Previous PVI, n (%) | 7 (13) | 11 (8) | 0.3 |
| Previous PVI + CTI, n (%) | 2 (4) | 7 (5) | 1 |
| Previous complex ablation procedure, n (%) | 29 (56) | 94 (65) | 0.2 |
| PVI with PV trigger, n (%) | 14 (27) | 30 (21) | 0.4 |
| Chronic heart failure, n (%) | 4 (8) | 8 (6) | 0.7 |
| Hypertension, n (%) | 41 (79) | 102 (72) | 0.34 |
| Diabetes mellitus, n (%) | 4 (8) | 16 (11) | 0.6 |
| Prior stroke, n (%) | 4 (8) | 12 (8) | 1 |
| CHA2DS2-VASc Score, mean ± SD | 2.69 ± 1.42 (52) | 2.82 ± 1.61 (142) | 0.6 |
| Coronary artery disease, n (%) | 9 (17) | 33 (23) | 0.4 |
| Antiarrhythmic drug, n (%) |  |  |  |
| Flecainide, n (%) | 3 (6) | 7 (5) | 0.7 |
| Amiodarone, n (%) | 11 (21) | 24 (17) | 0.5 |
| Dronedarone, n (%) | 1 (2) | 3 (2) | 1 |
| Propafenone, n (%) | 0 (0) | 1 (1) | - |
| Betablocker, n (%) | 39 (75) | 112 (79) | 0.5 |

**Supplement Table 2: Procedure characteristics of patients with an early recurrence vs. patients without an early recurrence**

| Baseline characteristics (N= 194) | Pts. with ER (N=52) | Pts. without ER (N=142) | p |
| --- | --- | --- | --- |
| Total procedure time min, mean ± SD (n) | 112.56 ± 38 (52) | 107.13 ± 38.12 (141) | 0.4 |
| Fluoroscopy time min, mean ± SD (n) | 15.35 ± 11.19 (52) | 14.19 ± 9.02 (141) | 0.7 |
| Rhythm at baseline |  |  |  |
| Clinical AT, n (%) | 35 (67) | 107 (75) | 0.35 |
| AF, n (%) | 2 (1) | 2 (1) | 0.5 |
| Sinus rhythm, n (%) | 15 (25) | 33 (22) | 0.697 |
| Mechanism of clinical AT |  |  |  |
| Macro-reentrant, n (%) | 35 (67) | 81 (57) | 0.3 |
| Focal, n (%) | 13 (25) | 46 (32) | 0.4 |
| Localized reentry, n (%) | 4 (8) | 15 (11) | 0.8 |
| Localization |  |  |  |
| Left, n (%) | 42 (81) | 116 (82) | 1 |
| Right, n (%) | 6 (12) | 15 (11) | 0.79 |
| Biatrial, n (%) | 4 (8) | 11 (8) | 1 |
| Ablation |  |  |  |
| Pulmonary vein re-isolation, n (%) | 15 (29) | 53 (37) | 0.31 |
| Mitral isthmus line, n (%) | 15 (29) | 48 (33) | 0.6 |
| Anterior line, n (%) | 14 (27) | 35 (25) | 0.6 |
| Roof line, n (%) | 20 (38) | 50 (35) | 0.4 |
| Cavotriscuspid isthmus, n (%) | 12 (23) | 38 (27) | 0.8 |
| prior substrate modification, n (%) | 26 (50) | 82 (58) | 0.4 |

**Supplement Table 3: Analyses adjusted for age, sex, new use of antiarrhythmic drugs and type of index procedure (non-complex vs. complex AF ablation )**

| **Variable** | **Hazard-ratio** | **95%-CI** | **p-value** | **Significance** |
| --- | --- | --- | --- | --- |
| Early Recurrence | 1.58 | 1.04 – 2.4 | 0.03 | * |
| Age (years) | 0.98 | 0.98 –1.01 | 0.09 |  |
| Sex (Male) | 0.74 | 0.75 – 1.34 | 0.17 |  |
| Non-complex vs. complex AF ablation | 1.32 | 0.89 – 1.96 | 0.17 |  |
| New AAD use | 0.85 | 0.86 – 1.16 | 0.64 |  |

**Supplement figure 1: High Density maps of macro-reentry, focal and micro-reentry atrial tachycardias**

Panel A: macro-reentry atrial tachycardia (AT) involving the posterior wall. The propagation wavefront originates at the early-meets-late region. Panel B: Focal atrial tachycardia with the earliest activation (red color) arising from the lateral left atrial wall. Panel C: Micro-reentry AT at the anterior aspect of the right superior pulmonary vein, characterized by long, fractionated electrograms at the site of the earliest activation.
